# A review of the role of cav-1 in neuropathology and neural recovery after ischemic stroke

**DOI:** 10.1186/s12974-018-1387-y

**Published:** 2018-12-20

**Authors:** Qianyi Huang, Wei Zhong, Zhiping Hu, Xiangqi Tang

**Affiliations:** Department of Neurology, The Second Xiangya Hospital, Central South University, Renmin Road 139#, Changsha, 410011 Hunan China

**Keywords:** Ischemic stroke, Cav-1, BBB permeability, Neurogenesis, Angiogenesis, Neuroinflammation, Apoptosis, Oxidative stress

## Abstract

Ischemic stroke starts a series of pathophysiological processes that cause brain injury. Caveolin-1 (cav-1) is an integrated protein and locates at the caveolar membrane. It has been demonstrated that cav-1 can protect blood–brain barrier (BBB) integrity by inhibiting matrix metalloproteases (MMPs) which degrade tight junction proteins. This article reviews recent developments in understanding the mechanisms underlying BBB dysfunction, neuroinflammation, and oxidative stress after ischemic stroke, and focuses on how cav-1 modulates a series of activities after ischemic stroke. In general, cav-1 reduces BBB permeability mainly by downregulating MMP9, reduces neuroinflammation through influencing cytokines and inflammatory cells, promotes nerve regeneration and angiogenesis via cav-1/VEGF pathway, reduces apoptosis, and reduces the damage mediated by oxidative stress. In addition, we also summarize some experimental results that are contrary to the above and explore possible reasons for these differences.

## Introduction

Stroke is a common cerebrovascular event and is one of the leading causes of mortality and morbidity worldwide. Ischemic stroke (IS) and hemorrhagic stroke are the two major categories of stroke, and IS is more common, accounting for 87% of all strokes [1]. IS is caused by a blocked blood vessel as a result of a thrombus or embolus and leads to hypoxia and loss of glucose in the cerebral tissue that survives [2]. Intensive basic and clinical studies have revealed a variety of stroke risk factors and elucidated many mechanisms of ischemic brain injury. Cerebral ischemia initiates multiple pathophysiological processes, including vasogenic edema, excitotoxicity, disruption of the blood–brain barrier (BBB), oxidative stress, cerebral inflammation, and neuronal death [3, 4]. Although significant progress has been made in understanding the pathophysiology of IS, patients with acute brain ischemia still lack treatment options.

Administration of tissue plasminogen activator (tPA) is the most effective treatment option for cerebral ischemia several years ago [5], but its use is limited to a narrow window after the onset of stroke, as the risk of hemorrhagic transformation increases over time, causing increased brain damage. Since 2015, mechanical thrombectomy has become the first-line treatment for anterior circulation stroke with proximal large-artery occlusion. However, only about 20% of stroke patients have large-artery occlusion and it is a challenge to deliver treatment to them within the 24-h time window because the procedure can only be performed in highly specialized centers [6]. In general, for more patients who cannot accept thrombolysis or mechanical thrombectomy, it is essential to elucidate the molecular mechanisms underlying IS and explore potential therapeutic targets to restore function after stroke.

The caveolae of the cell membrane are invaginations of the plasma membrane with an omega shape and a diameter of 60–80 nm which are rich in cholesterol and glycosphingolipids [7]. The caveolin family of proteins, which includes caveolin-1 (cav-1), caveolin-2, and caveolin-3, are located in cell membrane caveolae [8]. Cav-1 is the major component, is a specific marker of caveolae, and is generally distributed in smooth muscle cells, endothelial cells, skeletal myoblasts, fibroblasts, and adipocytes [9]. Cav-1 modulates a wide range of cellular events such as proliferation, lipid metabolism, cellular tracking, and signal transduction [10]. Cav-1 has also demonstrated a beneficial role in IS. In this review, we discuss how caveolins modulate BBB permeability and the relationships between cav-1 and a series of processes including neurogenesis, angiogenesis, neuroinflammation, apoptosis, and oxidative stress in IS.

## Pathophysiological mechanisms in IS: BBB damage, inflammation, and free radicals

In acute IS, an embolic or thrombotic event usually results in a rapid reduction in blood supply to a specific brain region, temporarily or permanently. The glucose and oxygen supply to the brain decreases, causing a rapid decline in ATP and a subsequent large movement of Ca^2+^ from the extracellular to the intracellular space [11]. Because of a lack of energy, the membrane ion pump which includes the Na^+^-K^+^-ATPase fails to efflux intracellular sodium and this is a fundamental reason for cytotoxic edema [12]. BBB damage further exacerbates brain edema and cerebral injury [13]. Middle cerebral artery occlusion (MCAO) with reperfusion leads to a biphasic disruption of the BBB [14]. The initial opening is reversible and is associated with the activation of MMP-2. The second opening of the BBB is mediated by MMP-3 and MMP-9 which are induced by inflammatory cytokines [15]. Matrix metalloproteases (MMPs) are a group of endopeptidases that cleave protein substrates including fibronectin, laminin, proteoglycans, and type-IV collagen based on Zn^2+^ ion [16]. The early degradation of tight junction (TJ) proteins appears to be associated with MMP-2 in the early period of ischemia, and direct injection of MMP-2 into the rat brain disrupts the BBB [17]. However, MMP-2 knockout did not provide neuroprotection in a rodent MCAO stroke model [18]. This may indicate that MMP-2 is not the major deleterious enzyme in the MMP response to ischemic stroke. In contrast, knockout of the MMP-9 gene demonstrates significant neuroprotection in an MCAO animal model [19]. Although inhibition or knockout of MMP-9 attenuates early degradation of the BBB in MCAO models, it is ineffective in preventing later opening of the BBB at 48 h after IS, and MMP-9 is thought to be beneficial in later stroke recovery, especially in promoting angiogenesis and neurogenesis [8, 20]. Therefore, it remains a challenge to identify agents that restore the integrity of the BBB and prevent brain edema without intervening in recovery.

Post-ischemic inflammation is another critical factor in the evolution of cerebral damage in IS models. In the first few hours after IS, microglial cells resident in the brain are activated and release toxic proinflammatory cytokines. These cytokines enable leukocytes to transmigrate across the endothelium and exacerbate brain infarction [21, 22]. Following microglial activation, peripheral macrophages, lymphocytes, and dendritic cells migrate to the site of injury, which precedes a neutrophil influx [23]. The infiltrating neutrophils promote BBB breakdown, resulting in deteriorating stroke outcomes. Inhibiting the upregulation of neutrophil integrins can ameliorate inflammatory responses and BBB dysfunction after ischemia [24]. It has been shown that neutrophils in the infarct core produce MMP-9 after IS and MMP-9 can further promote leukocyte recruitment. This positive feedback contributes to serious BBB breakdown and neuronal injury [25]. In terms of lymphocytes, Yilmaz et al. has shown that CD4^+^ (helper T cells, Th) and CD8^+^ T cells (cytotoxic T cells, Tc) are promotors of brain infarction and contributors to inflammatory responses after IS [26]. Previous studies have found that CD4^+^ Th1 cells secrete pro-inflammatory cytokines, such as interferon gamma, and lymphotoxin alpha in stroke, whereas CD4^+^ Th2 produce anti-inflammatory cytokines including IL-4, IL-10, and IL-13 [27]. Contrary to Th cells, the role of regulatory cells (Tregs) after ischemia is controversial. Liesz et al. found an increase in delayed brain damage in Treg-deficient mice, but less secondary infarct growth after the re-expression of Treg cells [28]. However, Kleinschnitz et al. showed that knockout of endogenous Tregs can reduce the cerebral volume of infarction and improve functional outcomes [29]. Another study has clearly demonstrated that depletion of B cells profoundly increases infarct volumes and mortality and impairs neurological function [30].

Free radicals and reactive species are thought to play major roles in cerebral ischemia reperfusion (I/R) injury. Reactive species include reactive oxygen species (ROS) and reactive nitrogen species (RNS). Huge quantities of ROS are produced following ischemia reperfusion, and they can increase brain damage through different mechanisms. For instance, ROS can destroy some cellular macromolecules contributing to autophagy, apoptosis, and necrosis [31]. ROS enhance inflammatory responses by activating inflammation factors and promoting leucocyte infiltration [32]. Furthermore, free radicals can affect BBB permeability through different ways. For example, ROS can oxidize and peroxidize proteins and lipids, and peroxidation of membrane lipids can directly damage BBB integrity [33]. ROS can also activate ProMMPs which degrade TJ proteins, further inducing BBB hyperpermeability. Reduction of ROS through the knockdown of NADPH oxidase blocks the upregulation of MMP-9 by inhibiting nuclear factor-κB (NF-κB)-dependent MMP-9 promoter activity [34]. ROS produced by the xanthine/xanthine oxidase system can directly redistribute TJ proteins by acting on the Rho, PI3K, and PKB pathways [35]. RNS, typically including NO and peroxynitrite, mediate BBB damage and functional deficits following IS. Physiological concentrations of NO are essential for a variety of processes, including neuronal communication, vascular tone regulation, and synaptic transmission [36, 37]. High concentrations of NO can induce inflammation and apoptotic cell death, resulting in a larger infarction size, which is detrimental to the ischemic brain tissue [38, 39]. RNS also mediate MMP activation in cerebral I/R injury [40]. Thus, reducing free radicals could be an effective mechanism for protection in acute ischemic stroke.

## Caveolae and caveolins

Caveolae were first observed by Palade and Yamada independently in the 1950s [41]. Caveolins and cavins have been found to be critical for the caveolae formation. Cavin, which is also termed polymerase I and transcript release factor, is an adapter protein that forms oligomers and assists in membrane curvature [42]. Caveolins are 22–24 kDa integral membrane proteins which are divided into three groups, caveolin-1, caveolin-2, and caveolin-3, with an NH_2_-terminal membrane attachment domain and a COOH-terminal membrane attachment domain that bind to membranes with high affinity, and both the N- and C-termini face the cytoplasm [43, 44]. Cav-1 and cav-2 are ubiquitously distributed, while cav-3 is primarily expressed in vascular smooth muscle, skeletal, and cardiac muscle cells. However, cav-3 has also been detected in astrocytes, neurons, and microglial cells [45, 46]. In the brain, cav-1 and cav-2 are primarily expressed in endothelial cells, and cav-3 is expressed in astrocytes [47, 48]. Human cav-1 and human cav-3 share 65% sequence identity and 85% sequence similarity and display similar activities [49]. Cav-2 shares 38% sequence identity and 58% sequence similarity with cav-1 [50], but unlike cav-1, cav-2 is not essential for caveolae formation. In cav-2-deficient mice, although the expression of cav-1 is decreased, the formation of caveolae is not affected [51]. Caveolins contain several separate domains: an N-terminal domain (residues 1–53), a caveolin-scaffolding domain (CSD) (residues 54–73), a transmembrane domain (residues 74–106), and a C-terminal domain (residues 107–151) [52]. Cav-1 and cav-3 interact with many proteins through the CSD. The CSD binds to caveolin-binding motifs (CBDs). This binding motif is characterized by the amino acid sequence: ΦXΦXXXXΦ, ΦXXXXΦXXΦ, ΦXΦXXXXΦXXΦ, where Φ is an aromatic residue, such as tyrosine, tryptophan, or phenylalanine, and X is any amino acid [53]. The CSD functions as a docking site for many intracellular signaling proteins. For example, CBD binding to the CSD usually inhibits a diverse range of proteins, such as eNOS, epithelial growth factor receptor, PKA, PKC, G proteins, and Src family proteins. Moreover, the CSD has also been described as an activator of insulin receptor signaling [54, 55].

Functionally, caveolae are important in endocytosis, oncogenesis, and phagocytosis [56]. Caveolins can bind and regulate proteins with the CBD including NOS, MMP2, and epidermal growth factor receptor (EGFR), and they are involved in numerous cellular activities such as apoptosis [57], cholesterol transport [58], and cancer metastasis [59, 60]. Cav-1, a well-established major structural protein of caveolae, has been suggested as playing an important role in the regulation of multiple cellular processes, including cell growth, differentiation, endocytosis, cholesterol trafficking, and cellular senescence [61]. Interestingly, it has been shown that cav-1 regulates the anti-atherogenic properties of macrophage, but cav-1 promotes atherosclerosis in endothelial cells [62, 63]. In the cerebrum, cav-1 regulates neuronal signaling and promotes dendritic growth [64]. Changes in cav-1 can sequentially induce a series of alterations in BBB permeability, neuroinflammation, cerebral angiogenesis, apoptosis, and oxidative stress, which we will discuss in detail.

## Important role of cav-1 in IS

Recently, it has been shown that cav-1 has a beneficial role in cerebral ischemia. Overexpression (OE) of cav-1 decreased brain edema following photothrombosis and MCAO in rats [65]. Knockout (KO) of the cav-1-encoding gene in mice produced an enlargement in infarction size, impaired angiogenesis, and increased apoptotic cell death compared with wild-type (cav-1(+/+)) mice and cav-2-deficient (cav-2(−/−)) mice [66]. Recently, Choi et al. found that cav-1 KO mice showed a dramatic increase in the extent of BBB disruption compared with wild-type mice, and this effect was inhibited by lentiviral-mediated re-expression of cav-1 [67]. However, some researchers believe that the protective effects in brain ischemia of several natural active compounds are related to the reduced expression of cav-1. Zhang et al. showed that green tea polyphenols could reduce BBB permeability and brain edema, and this neuroprotection effect may be related to the downregulation of cav-1 and phosphorylated ERK1/2 [68]. Huang et al. also found that post-infarct treatment with Cerebralcare Granule significantly decreased the elevated BBB permeability in the ischemic region, which was associated with the inhibition of cav-1 in the endothelial cells [69]. The discrepancy is probably due to the multifaceted role of natural objects and different stroke models. Their protective role in ischemic stroke is not necessarily due to the reduction of cav-1, and there is no further intervention on cav-1. Further studies are still needed to confirm if reduced cav-1 is one of the mechanisms of the protective effects of natural compounds. The following discussion is about the role of cav-1 in ischemic stroke.

### Cav-1 regulates BBB permeability in stroke (Fig. 1)

It is well established that cav-1 is closely related to BBB permeability in stroke. Experimental models of cerebral ischemia indicate that the downregulation of cav-1 membrane protein results in increased BBB permeability [70]. In a clinical study, low serum levels of caveolin-1 were considered to be a predictor of symptomatic hemorrhagic transformation (sHT). sHT is related to increased endothelial permeability after r-tPA administration [71].

**Fig. 1:**
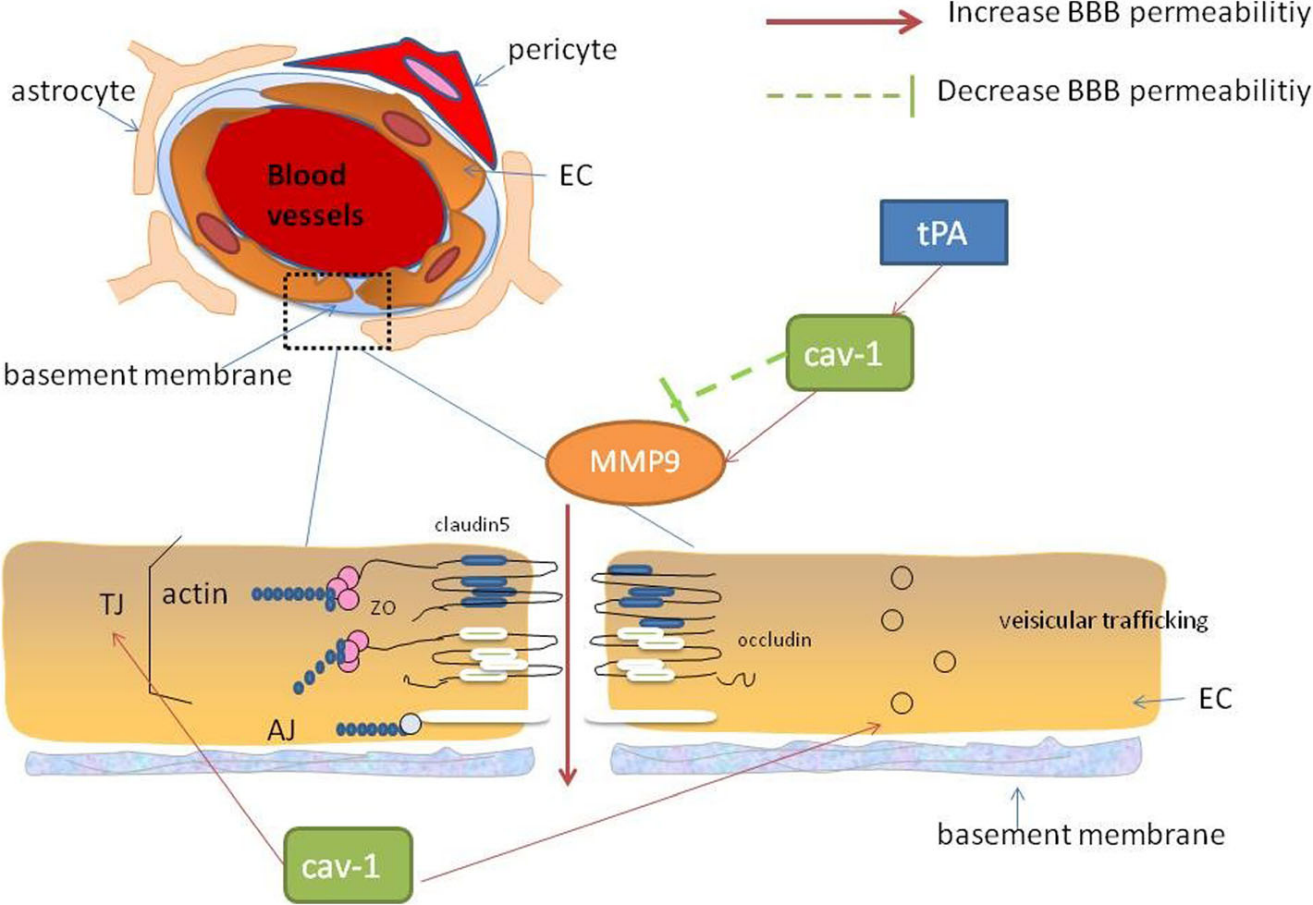
Effect of caveolin-1 (cav-1) on the blood-brain barrier (BBB). Cav-1 increases the BBB permeability via caveolae-based transcytosis and translocation of tight junction (TJ) protein. Cav-1 inhibits matrix metalloproteinase-9 (MMP-9) which disrupts TJ proteins and basement membrane under ischemic stroke condition, while cav-1 appears to promote MMP-9 upregulation by tPA. However, cav-1 (−/−) mice demonstrated higher MMP activity and BBB permeability than cav-1(+/+) mice in a focal cerebral ischemia-reperfusion model, which means that cav-1 may mainly protects BBB integrity after IS. Endothelial cell (EC), ischemic stroke (IS), tight junction (TJ): actin ZO claudin 5 occludin

Cav-1 predominantly regulates BBB permeability through transcellular and paracellular routes. There are 14-fold fewer vesicles in the brain endothelium than in the endothelium of non-neural vessels, which demonstrates the unique properties of central nervous system (CNS) endothelial cells: their limited vesicular transport (transcytosis). Numerous macromolecules, including albumin, lipoproteins, insulin, and transferrin, have been shown to be transendothelially delivered through caveolae [72]. *Phoneutria nigriventer* spider venom-induced BBB opening is relevant to increased expression of cav-1α [73]. Cav-1 KO mice showed defects in the uptake and transport of albumin from the blood to the interstitium [74]. Co-administration of focused ultrasound and a dose of microbubbles resulted in a high expression of cav-1 and increased BBB permeability through a caveolae-mediated transcellular mechanism [75]. Feng et al. demonstrated that vascular endothelial growth factor (VEGF) enhanced retinal endothelial cell permeability via eNOS-dependent transcytosis in caveolae [76]. Knowland et al. showed that the increased endothelial caveolae number and transcytosis rate account for early BBB hyperpermeability after MCAO [77]. Therefore, it is considered that cav-1 can increase the BBB permeability via caveolae-based transcytosis.

Caveolae-mediated transport is regulated by Src-mediated phosphorylation. To regulate caveola formation and fission, it is essential to orchestrate the localization and activity of proteins of the endocytic machinery [78]. Src-mediated phosphorylation of caveolin-1 at Tyr14 initiates plasmalemmal vesicle fission and transendothelial vesicular transport [79]. Sun et al. observed that phosphorylation of cav-1 is crucial in H_2_O_2_ exposure-induced pulmonary vascular hyperpermeability, which occurs through transcellular and paracellular pathways [80]. In terms of the regulatory mechanism of cav-1 phosphorylation, Takeuchi et al. showed that oxidative stress-induced cav-1 phosphorylation and endocytosis was inhibited by the activation of AMPK, in part by suppressing the dissociation between c-Abl and Prdx1 proteins [81]. Andreone et al. reported the regulation of CNS endothelial cell lipid composition specifically inhibited the caveolae-mediated transcytosis [82].

Decreased levels of TJs typically increase BBB permeability. Cav-1 affects paracellular permeability by controlling MMPs. Cav-1-deficient mice showed higher MMP proteolytic activity and breakdown of TJ proteins than wild type mice, and the opposite effects were observed following re-expression of cav-1 [67]. Downregulation of cav-1 led to decreased expression of TJ-associated proteins, proteolysis of TJs, and opening of the blood–tumor barrier (BTB), whereas cav-1 OE restored the expression of TJ-associated proteins [83]. Gu et al. found that cav-1 in the ischemic region was reduced after focal cerebral ischemia with reperfusion. The decreased cav-1 was associated with enhanced activities of MMP-2 and MMP-9 activity, downregulation of zonula occludens-1 expression, and increased BBB permeability. The researchers used two methods, including cav-1 knockdown in cultured brain microvascular endothelial cells (BMECs) and a cav-1 gene KO mouse model. Cav-1 knockdown resulted in high MMP activity and BMEC hyperpermeability. Cav-1(−/−) mice demonstrated higher MMP activity and BBB permeability than cav-1 (+/+) mice in a focal cerebral ischemia-reperfusion model [70]. Interestingly, cav-1 seems to play a role in tPA-induced MMP-9 activation in the ischemic brain, which exaggerates brain infarction and increases the risk of symptomatic cerebral hemorrhage. To determine whether cav-1 is involved in tPA-induced MMP-9 upregulation, cultured BMECs were treated using cav-1 siRNA. It was shown that knockdown of cav-1 blocked tPA-induced MMP-9 mRNA increases and increases in MMP-9 protein levels in the conditioned media, but did not affect decreased MMP-9 levels in cellular extracts. It means that cav-1 may not affect MMP-9 secretion from the endothelial cells [84]. However, Song et al. reported that cav-1 s-nitrosylation is involved in the secretion of MMP-2 and MMP-9 from tPA-treated ischemic endothelial cells. In the context of tPA-induced ERK activation, oxygen-glucose deprivation (OGD)-triggered cav-1 s-nitrosylation can enhance MMP-2 and MMP-9 secretion and subsequently promote extracellular matrix (ECM) degradation [85]. Taken together, cav-1 inhibits MMP-9 under ischemic stroke condition. In contrast, cav-1 appears to promote MMP-9 upregulation by tPA. These findings give us a hint that the effect of cav-1 may be stimulus-dependent. And more work is needed to clarify the signaling mechanisms.

Cav-1 is also connected with the distribution of TJ proteins. Liu et al. found that MMP-2 was responsible for OGD-induced occludin degradation, and in vitro, cav-1 mediated claudin-5 redistribution in OGD [86]. They further investigated the fate of translocated claudin-5 and the mechanisms through which OGD promoted cav-1 translocation, and their data demonstrate that NO promotes the delivery of claudin-5 to the autophagosome for autophagy-lysosome-dependent degradation which is mediated by cav-1 [87]. Caveolae also mediate the internalization of TJ proteins, including occludin and claudin-5, when brain endothelial cells are exposed to chemokine (C-C motif) ligand 2 (CCL2) which induces TJ remodeling [88].

Contrary to the view that cav-1 is neuroprotective in cerebral ischemia, there are a host of studies demonstrating that cav-1 may damage the BBB. To simulate this situation, three models of high BBB permeability were established, including MCAO insult, lipopolysaccharide (LPS) treatment, and cold injury. In these models, the expression of both claudin-5 and VE-cadherin was reduced, whereas cav-1 protein expression was increased [89]. In rats with cerebral ischemia-reperfusion injury, Xie et al. demonstrated that inhibiting transient receptor potential vanilloid 4 ameliorated BBB disruption through reducing the expression of cav-1 and cav-2 [90]. Pretreatment with electroacupuncture profoundly ameliorated the elevated BBB permeability and reduced brain edema in a focal I/R rat model, and it was related to the decreased degradation of TJ proteins and downregulation of the expression of p-cav-1 in endothelial cells [91]. The discrepancy in previous studies may arise from different ischemia models, different time of ischemia-reperfusion, or different sample sources. In general, cav-1 can increase BBB permeability through caveolae-mediated endocytosis and translocation of TJ protein, but can also protect BBB integrity by inhibiting MMP activity during ischemic stroke.

### Cav-1 and angiogenesis (Fig. 2)

Angiogenesis refers to the sprouting of new microvessels from existing vasculature. It is a process of endothelial proliferation, migration, and differentiation and is often stimulated by hypoxia [92]. Both in humans and rodents, angiogenesis takes place in the area surrounding a brain infarction, starting 3–4 days after the ischemic insult [93, 94]. Upon the onset of ischemia, NO and VEGF lead to vasodilation and increased vascular permeability, resulting in extravasation of plasma proteins. Subsequently, these proteins lay down a temporary scaffold for the migration of endothelial cells which is necessary for vascular sprouting [95], and then angiopoietin-2 dissociates smooth muscle cells and loosens the ECM [96]. Following complete restructuring of the ECM, ECM tracts are formed [92]. ECM tracts are used to establish new capillary buds. Angiogenesis is tightly controlled through a balance between angiogenic (pro-angiogenic) and angiostatic (anti-angiogenic) factors. VEGF is considered to be the major angiogenic factor [97]. Activation of the vascular endothelial growth factor receptor 1 (VEGFR-1) and VEGFR-2 promotes endothelial cell proliferation and migration and exacerbates ECM degradation through different mechanisms including the PI3K/Akt and MEK/ERK protein kinase pathways [98]. NO, miRNAs, and Ang also have important roles in mediating angiogenesis. NO can increase the expression of VEGF in endothelial cells and increase vascular permeability in the ischemic brain. Ang can stabilize and remodel nascent blood vessels by binding to Tie-2 receptors. miR-210 and miR-140-5p modulate angiogenesis via upregulation of VEGFA expression, whereas it has been reported that miR-15a and miR-150 reduced vascular density in the infarct region in a rat model of MCAO [98].

**Fig. 2:**
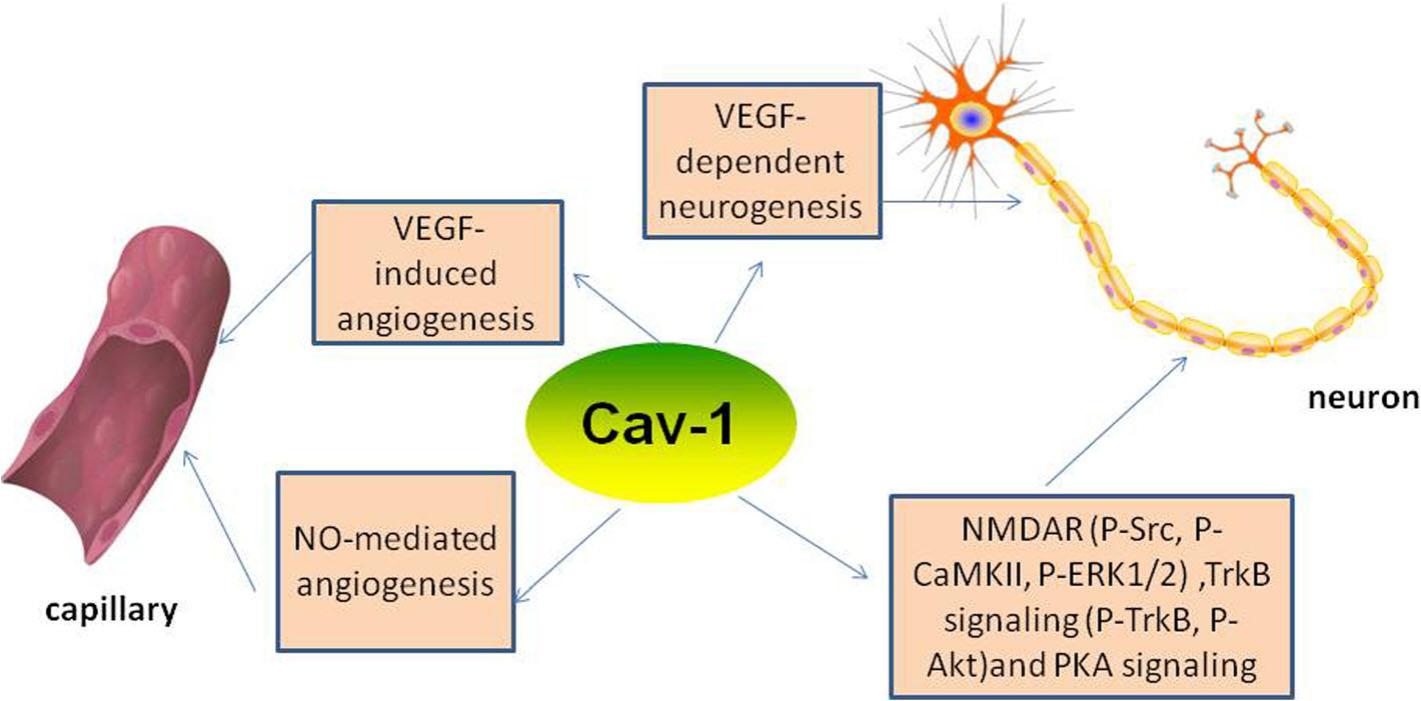
The role of cav-1 in angiogenesis and neurogenesis. Cav-1 is essential for NO-mediated angiogenesis and VEGF-induced angiogenesis. VEGF can promote NO production and NO can increase the expression of VEGF. Cav-1/VEGF-dependent pathway is important for neurogenesis, and cav-1 enhances NMDAR (P-Src, P-CaMKII, P-ERK1/2), TrkB signaling (P-TrkB, P-Akt), and PKA signaling, and augments dendritic growth and arborization. Methyl-D-aspartate receptor (NMDAR), calcium/calmodulin-dependent protein kinase II (CaMKII), phosphorylated (P)-extracellular signal-regulated kinase (ERK), tyrosine kinase B receptor (TrkB), cAMP-dependent protein kinase (PKA)

Treadmill exercise has been demonstrated to promote angiogenesis in the infarct penumbra of animal brains, and an exercise group showed significantly higher expression of cav-1 and VEGF protein, better neurological recovery, and a smaller cerebral volume of infarction than a non-exercise group. And rats received the injection of the cav-1 inhibitor, daidzein, showed decreased expression of VEGF compared with the exercise group, which probably means that cav-1 pathway is involved in the regulation of VEGF after treadmill exercise [99]. Treatment using apigenin upregulated the expression of cav-1, VEGF, and eNOS compared with a control group, and cav-1 silencing counteracted apigenin-induced angiogenesis in vitro by promoting the tube formation ability of human umbilical vein endothelial cells (HUVECs) [100]. Genetic ablation of the cav-1 in mice resulted in impaired angiogenesis and increased apoptotic cell death in the ischemic brain. In vitro, adenoviral-mediated OE of caveolin-1 clearly accelerated endothelial cell differentiation/tubule formation and led to an upregulation in the number of capillary-like tubular structures. Conversely, knockdown of cav-1 using an antisense adenoviral gene delivery system reduced the number of capillary-like tubules, which demonstrates that cav-1 plays a positive role in the regulation of endothelial cell differentiation [101]. These findings suggest that cav-1 may promote angiogenesis in IS. More specifically, in the process of angiogenesis, it appears that cav-1 negatively regulates endothelial cell proliferation and promotes cell differentiation [79]. Cav-1 inhibited endothelial cell proliferation by arresting cell cycle at the G0/G1 phase [102]. PD98059 is a specific inhibitor of mitogen-activated protein kinase and can suppress angiogenesis. PD98059 can also block decreases in cav-1 expression. Therefore, it is quite reasonable to consider that decreased cav-1 expression may play a significant role in the signaling required for endothelial cell proliferation [103].

Cav-1 has been shown to interact with certain angiogenic (pro-angiogenic) factors. Treatment of a HUVEC line (ECV 304) with some well-known angiogenic growth factors (VEGF, basic fibroblast growth factor, or hepatocyte growth factor/scatter factor) contributed to a dramatic reduction in cav-1 expression [103]. VEGFR-2 is localized in endothelial caveolae, and OE of cav-1 inhibits the activity of VEGFR-2 [104]. However, cav-1 also activates VEGFR-2 via a VEGF-dependent pathway, and the removal of caveolin and VEGFR-2 from caveolae leads to the inhibition of endothelial cell migration [104]. Re-expression of cav-1 in cav-1-deficient endothelial cells resulted in the relocation of VEGFR-2 in caveolar membranes and restored the VEGF-induced ERK and eNOS activation [105]. Cav-1 is also essential for NO-mediated angiogenesis. VEGF promotes the production of NO and the formation of capillary-like tubules. Antisense cav-1 oligonucleotides were transfected into HUVECs, and this resulted in a significant decrease in capillary-like tubules, and NO production did not respond to the addition of VEGF and NG-nitro-L-arginine methyl ester (L-NAME), a nonselective NOS inhibitor [106]. Although some angiogenic growth factors inhibit cav-1, cav-1 is important for VEGF or NO-mediated-angiogenesis. Cav-1 is a potential therapeutic target to improve angiogenesis for the treatment of stroke.

### Cav-1 and neurogenesis (Fig. 2)

Similar to the angiogenic process, neurogenesis includes the proliferation, migration, and differentiation of neural stem cells (NSCs), which can differentiate into a variety of cells, including neurons, ependymal cells, astrocytes, and oligodendrocytes [107].

For instance, in neurogenesis in the olfactory bulb, initially, NSCs differentiate into neuroblasts, and then they migrate to the olfactory bulb and differentiate into periglomerular or granule neurons [108]. After stroke, if endogenous NSCs are not injured, NSCs will migrate to the ischemic region and differentiate into astrocytes [109]. Studies have demonstrated that adult NSCs in the subventricular zone (SVZ) of the lateral ventricle and the dentate gyrus of the hippocampus can be activated and produce neuroblasts which will recruit to the infarcted region and participate in repairing the ischemic brain tissue [110, 111]. The PI3K pathway, Wnt/beta-catenin pathway, the Notch pathway, and the Sonic Hedgehog pathway have been reported as affecting neurogenesis, as discussed in a review written by Koh and Park [107].

As mentioned earlier, treadmill exercise promotes angiogenesis in the infarcted penumbra via a cav-1/VEGF-dependent pathway. Recently, a research group observed that the upregulation of both cav-1 and VEGF protein expression following treadmill exercise was consistent with improved recovery in neurological function which correlated with the proliferation, migration, and differentiation of SVZ-derived NSCs in the brain tissue. Furthermore, knockdown of cav-1 by a cav-1 inhibitor remarkably inhibited the treadmill exercise-induced upregulation in VEGF expression, improved neurological recovery, and decrease in the cerebral volume of infarction [112]. In addition, the cav-1/VEGF signaling pathway was found to play a role in basic fibroblast growth factor-induced angiogenesis and neurogenesis following treadmill exercise after acute IS [113]. This evidence suggests that cav-1 plays a positive role in neurogenesis. However, Li et al. found that in the hippocampal dentate gyrus (DG), cav-1 (−/−) mice displayed significantly higher levels of VEGF expression and more abundant formation of newborn neurons than cav-1 (+/+) mice. Cav-1 peptide markedly inhibited neuronal differentiation [114]. It has been reported that cav-1 can regulate synaptic plasticity. Decreased expression of hippocampal caveolin-1 caused synaptic plasticity deficits in aged rats [115]. Cav-1 knockdown inhibited the morphine-induced upregulation of neurite outgrowth [116]. Neuron-targeted cav-1 overexpression enhanced dendritic arborization within the apical dendrites of hippocampal and granule cell neurons in adult and aged mice [117]. Similarly, Egawa et al. showed that neuron-targeted OE of cav-1 (SynCav1) promoted ultrastructural and functional hippocampal synaptic plasticity [118]. Head et al. showed that cav-1 OE, achieved using a synapsin-driven cav-1 vector, upregulated the formation of lipid rafts and the expression of neurotransmitter and neurotrophin receptors in primary neurons [119]. In conclusion, cav-1 promotes neurogenesis and neuroplasticity after ischemic stroke, but some studies demonstrate that cav-1 inhibits neural progenitor cells differentiation under normoxic and hypoxic conditions through the downregulation of VEGF signaling. One feasible explanation could be the paradox phenomenon between cav-1 and VEGF: cav-1 can downregulate VEGF, but is also essential for VEGF signaling. The relationship between cav-1 and VEGF signaling still needs further work. In neurons, pro-survival signals occur after the activation of various synaptic signaling receptors, including the *N*-methyl-D-aspartate (NMDA) glutamate receptor (NMDAR), the neurotrophin-activated tyrosine kinase B receptor (TrkB), and G-protein coupled receptors. These survival signals converge via intracellular protein kinases (CaMKII, Src, Akt, cAMP) to phosphorylate extracellular signal-regulated kinase 1/2 (ERK1/2), initiating the transcription of pro-survival and pro-growth genes. These receptors are distributed in regions enriched in caveolins. Cav-1 OE enhanced NMDAR (P-Src, P-CaMKII, P-ERK1/2) and TrkB signaling (P-TrkB, P-Akt) and augmented dendritic growth and arborization [120]. On glial cell line-derived neurotrophic factor stimulation, expression of cav-1 is increased in mesencephalic dopaminergic neurons, and modulation of genes which are correlated with neuronal plasticity and survival is mediated in part by ERK and cAMP-dependent protein kinase (PKA) signaling [121]. Increased p-ERK1/2 was almost diminished by the inhibition of caveolin and vice versa [120]. Therefore, cav-1 is essential for ERK activation and appears to be important in pro-survival signaling. Thus, cav-1 could be a novel therapeutic target protein for promoting neurogenesis after ischemic stroke.

### Cav-1 and neuroinflammation (Fig. 3)

The role of caveolins in many aspects of the inflammatory response, including angiogenesis, leukocyte recruitment, pathogen invasion, the production of inflammatory mediators, and fibrosis, has been extensively demonstrated in the literature [122]. Severe BBB breakdown occurs several hours after ischemic attack and is followed by a massive infiltration of polymorphonuclear (PMN) leukocytes [123]. This event not only results in brain edema formation but also induces the activation of brain microglia which produce pro-inflammatory cytokines and ROS exacerbating the brain injury. Thus, we logically address that cav-1 inhibits inflammation via maintaining the integrity of BBB under brain ischemia condition. Niesman et al. illustrate that cav-1 regulates neuroinflammatory responses after traumatic brain injury (TBI), as cav-1 KO mice exhibited a significant increase in cytokine/chemokine production in a controlled cortical impact model of TBI [124]. Moreover, cavtratin, a cell permeable peptide of cav-1, has previously been shown to inhibit the transmigration and viability of macrophages and microglia via the JNK pathway and reduce inflammation in ocular neovascularization [125]. Cavtratin also reduces inflammatory cell infiltration, contributing to the improvement in BBB function in an immunologic mouse model of multiple sclerosis [126]. It is noteworthy that in healthy situation, cav-1 may as well inhibit inflammation, as systemic administration of cav-1 CSD to mice suppressed acute inflammation and vascular leak to the same extent as a glucocorticoid [127]. Caveolae have been reported as important in the internalization and later fate of several pathogens. For example, by targeting caveolae as an endocytic pathway, pathogens such as simian virus 40 and certain strains of *Escherichia coli* can be transported directly to the Golgi and/or ER, avoiding disruption in lysosomes and ensuring their intracellular survival [128]. However, cav-1 promotes the ability of macrophages to phagocytose both *E. coli* and apoptotic cells in vitro [129].

**Fig. 3:**
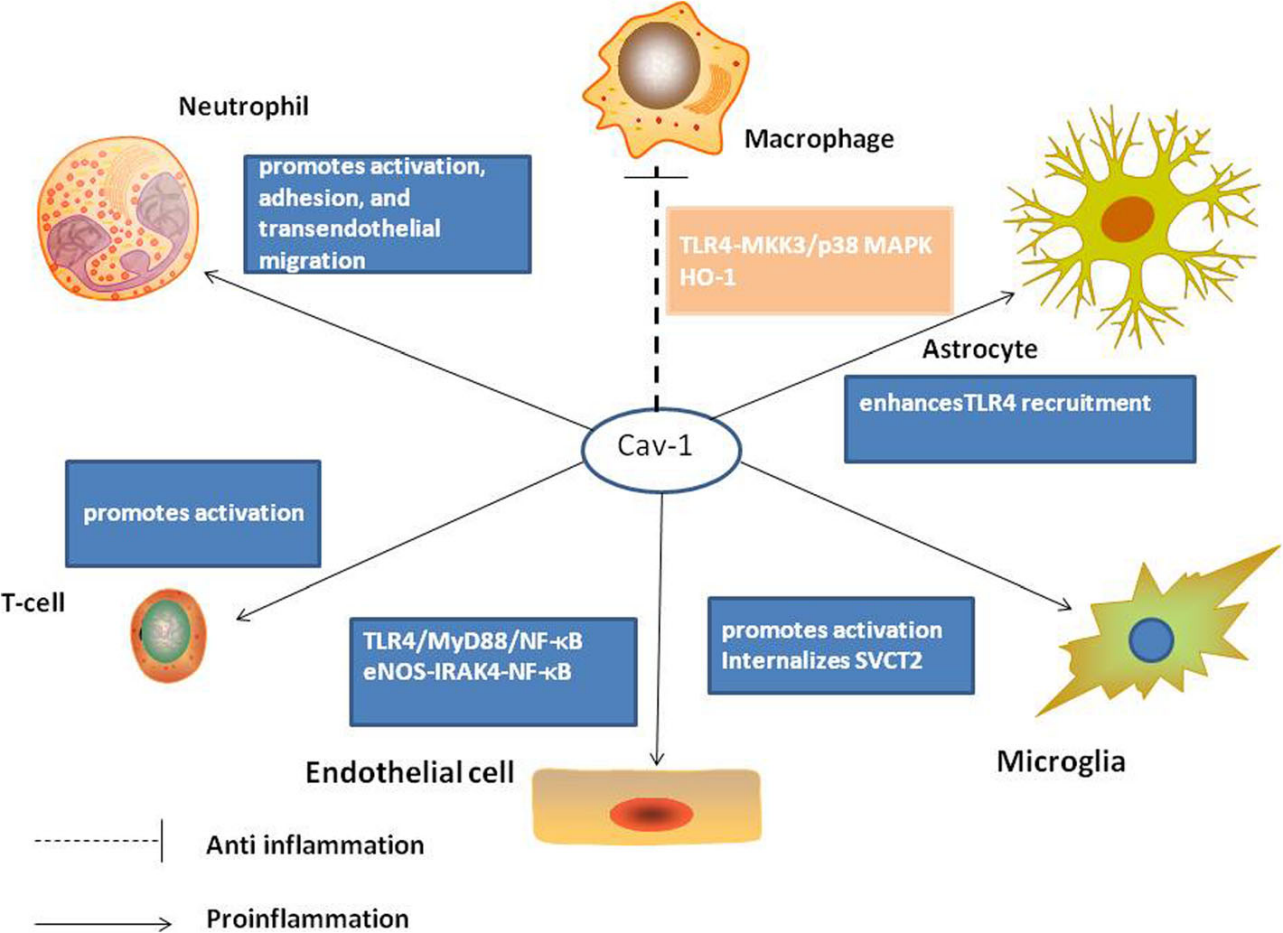
Schematic illustrating the role of cav-1 in different cell. In endothelial cells, after exposure to LPS, cav-1 interacts with toll-like receptor 4 (TLR4), and then TLR4 promotes activation of MyD88, leading to NF-κB activation and the generation of proinflammatory cytokines. Under normal condition, cav-1 inhibits eNOS. If eNOS is activated, NO will nitrate IRAK4 which is a signaling factor required for NF-κB activation. And cav-1 in microglia can induce the internalization of SVCT2 which is the primary mediator of vitamin C uptake in neurons, triggering a proinflammatory phenotype in microglia and inducing microglia activation. Whereas in macrophages, cav-1decreases proinflammatory cytokine production and augments anti-inflammatory production through the TLR4/MKK3/p38 MAPK pathway. Besides, TLR4 activation by LPS also stimulates HO-1 transporting to the caveolae and producing CO which has anti-inflammatory effects and augments the cav-1/TLR4 interaction in murine macrophages. Toll-like receptor 4 (TLR4), interleukin-1 receptor associated kinase 4 (IRAK4), heme oxygenase-1 (HO-1), carbon oxide (CO), plasma membrane sodium-vitamin C cotransporter 2 (SVCT2)

In the endothelium, after exposure to LPS, cav-1 was phosphorylated at Tyr(14) which resulted in interaction between cav-1 and toll-like receptor 4 (TLR4), and then TLR4 promoted activation of MyD88, leading to NF-κB activation and the generation of proinflammatory cytokines [130]. Cav-1 OE dose-dependently increased monocyte chemoattractant protein-1 (MCP-1), vascular cell adhesion molecule-1 (VCAM-1), and plasminogen activator inhibitor-1 (PAI-1) mRNA levels in human umbilical vein ECs. Pigment epithelium-derived factor binding to cav-1 could inhibit the pro-inflammatory effects of cav-1 in endothelial cells [131]. Cav-1 can affect NF-κB to influence the inflammatory response. Signaling by the proinflammatory cytokine interleukin-1β (IL-1β) is dependent on ROS, because after IL-1β binding to its receptor (IL-1R1), ROS generated by Nox2 is responsible for the downstream recruitment of IL-1R1 effectors (IRAK, TRAF6, IkappaB kinase kinases) and ultimately for activation of NF-κB. Lipid rafts and cav-1 coordinate IL-1β-dependent activation of NF-κB. Inhibiting cav-1-mediated endocytosis can prevent NF-κB activation [132]. Cav-1 gene KO lungs displayed suppression of NF-κB activity and reduced transcription of iNOS and intercellular adhesion molecule 1 (ICAM-1) [133]. Secondary to the loss of cav-1, eNOS is activated, which inhibits the innate immune response to LPS through interleukin-1 receptor-associated kinase 4 (IRAK4) nitration. IRAK4 is a signaling factor required for NF-κB activation and innate immunity. Cav-1 KO can impair the activity of NF-κB and reduce LPS-induced lung injury [134]. Cav-1 appears to have different roles in different cells. In macrophages, the interaction of cav-1 with TLR4 has an opposite outcome compared with that in endothelium, whereby overexpression of cav-1 decreased LPS-induced TNF-α and IL-6 proinflammatory cytokine production and augmented anti-inflammatory cytokine IL-10 production. Cav-1 regulates LPS-induced inflammatory cytokine production through the MKK3/p38 MAPK pathway [135]. It is valuable to mention that TLR4 activation by LPS also stimulates heme oxygenase-1 (HO-1) transporting to the caveolae by a p38 MAPK-dependent mechanism and producing carbon oxide (CO). CO has anti-inflammatory effects and augments the cav-1/TLR4 interaction in murine macrophages [136]. In PMN leukocytes, cav-1 appears to promote activation, adhesion, and transendothelial migration and shows a positive role in PMN activation-induced lung and vascular injury [137]. Cav-1 also plays a key role in antigen-presenting cells, leading to the activation of T lymphocytes [138]. Furthermore, apoptosis of thymocytes in cav-1-deficient mice was higher than that in wild type mice, indicating that cav-1 is a key regulator of thymocyte apoptosis during inflammation [139]. In addition, cav-1 has a possible role in microglial activation. Cav-1 was remarkably reduced and localized in the plasmalemma and cytoplasmic vesicles of inactive microglia, whereas active (amoeboid-shaped) microglia exhibited increased cav-1 expression [46]. Recently, Portugal et al. found that cav-1 in microglia induced the internalization of plasma membrane sodium-vitamin C cotransporter 2 (SVCT2), triggering a proinflammatory phenotype in microglia and inducing microglia activation [140]. Besides, cav-1 transfection into Fatty acid-binding proteins-KO astrocytes remarkably enhanced TLR4 recruitment into lipid raft and tumor necrosis factor-ɑ production after LPS stimulation [141]. Together, in non-pathological states or after LPS stimulation, cav-1 in endothelial cells increases proinflammatory cytokines, while cav-1 inhibits LPS-induced inflammatory effects in murine macrophages. Cav-1 is involved in activation of microglia, PMN, and T lymphocytes.

Apart from the LPS-eNOS-TLR4-NFκB, MKK3/p38 MAPK, and IL-1β-IL-1R1 pathways which we have already mentioned, cav-1 participates in additional inflammatory signaling pathways [142]. Cyclooxygenase (COX)-2 is pivotal in post-ischemic brain damage, and the selective inhibition of the COX-2 enzyme inhibited subsequent prostaglandin E2 (PGE2) production, ischemic BBB leakage, leukocyte infiltration, and edema formation [143]. In cav-1-null mice, COX-2 protein levels are higher. Cav-1 binds COX-2 in the endoplasmic reticulum (ER) and modulates its degradation through an ER-dependent mechanism [144]. This suggests that cav-1 can regulate inflammatory responses by controlling COX-2 expression.

### Cav-1 and apoptosis

Apoptosis is morphologically characterized by a programmed cell death [145]. However, necrosis is a process of physico-chemical cell destruction and involves cellular plasma membrane disruption [146]. Autophagy is mainly triggered by cell starvation, and cells orchestrate self-digestion of long-lived proteins and damaged organelles in the lysosome. Autophagy might principally serve for cell survival, but sometimes leads to cell death [147]. Apoptosis is a highly conserved cellular response to cellular stress, DNA-, or organelle damage, but also plays a crucial role in the removal of superfluous cells. The destruction of one cell can be beneficial: the removal of superfluous cells is necessary for the development of functioning organs, and the removal of injured or aged cells that have lost their function can help to maintain tissue vitality [148]. Apoptosis can be initiated via two main routes: the extrinsic and intrinsic pathways. The intrinsic apoptotic pathway is usually triggered by stimuli, including DNA damage, organelle dysfunction of either mitochondria or the ER, the accumulation of unfolded proteins, growth factor withdrawal, or hyperthermia. The extrinsic pathway is triggered when ligands bind their trans-membrane death receptors. The extrinsic pathway not only directly leads to the activation of proteases which cause cell death, but also promotes the release of pro-apoptotic factors from mitochondria, which are linked with the intrinsic pathway [148].

Caveolins are directly connected with both the intrinsic and extrinsic cell death pathways. The key to the extrinsic pathway is the expression of transmembrane receptors, called death receptors, including TNF-receptor 1, Fas/CD95, and the TNF-related apoptosis-inducing ligand receptors. A potential cav-1 binding motif (G53LHHDGQFCH) has been identified in the human death receptor Fas sequence [149]. Cav-1 KO disrupted death-induced signaling complex formation following colocalization and interaction between cav-1 and Fas [150]. Recently, Glukhova et al. found that the deletion of both caveolin-binding sites (which bind the N-terminal domain and B-terminal domain of cav-1) in the Fas-ligand attenuated extrinsic cell death pathway-associated cytotoxicity, demonstrating that the relationship between the Fas-ligand and cav-1 provides a molecular basis for the location of the ligand to lipid rafts and Fas-ligand-dependent apoptosis [151]. As for the intrinsic apoptosis pathway, mitochondrial outer membrane permeabilization is the central event of the intrinsic pathway: in response to apoptotic stimuli, mitochondria release intermembrane space proteins, such as cytochrome C and apoptosis-inducing factor, which exert a cryptic cytotoxic activity following their mitochondrial release [152]. And mitochondrial dysfunction is considered as a hallmark of intrinsic apoptosis. In E11 podocytes, cav-1 OE attenuated H_2_O_2_-induced oxidative stress responses and preserved mitochondrial function, as well as significantly suppressing apoptosis [153]. Moreover, various cav-1-deficient cells have displayed mitochondrial dysfunction including impaired energy generation, and increased mitochondrial ROS production [154–156]. Based on the abovementioned findings, we speculate that cav-1 could relay apoptotic signaling via allowing Fas multimerization, but also attenuates apoptosis through preventing mitochondrial dysfunction.

Additionally, cav-1 plays a different role in regulating apoptosis in different cell types. In HEK293T cells, cav-1 was shown to reduce survivin, which inhibits apoptosis through a pathway involving diminished beta-catenin-Tcf/Lef-dependent transcription. This suggests that cav-1 has pro-apoptotic properties [157]. However, there is also evidence to suggest that cav-1 suppresses apoptosis. In hypoxic human SK-N-MC neuroblastoma cells, iNOS-induced NO production reduces the expression of cav-1. OE of cav-1, either through cav-1 transfection or administration of a cav-1 CSD peptide, decreased the production of NO and impaired apoptotic cell death in hypoxic SK-N-MC cells [158]. Yue et al. demonstrated that cav-1 could suppress ER stress-induced macrophage apoptosis in vitro, and one of the underlying mechanisms may be associated with the activation of the p38 MAPK pro-survival pathway [159]. Cav-1 KO macrophages were more susceptible to apoptosis and more prone to induce inflammation than wild-type macrophages. Decreased cav-1 protein levels mediated I/R injury-induced liver damage by inhibiting sphingosine kinase 2/sphingosine-1-phosphate receptor 2 signaling and enhancing apoptosis [160].

The anti-apoptotic role of cav-1 in ischemic stroke is suggested by Jasmin et al.; they showed increased apoptotic cell death in cav-1 KO ischemic brains, as compared with WT ischemic brains. But the underlying mechanisms are still not clear. Recently, it was found that downregulation of cav-1 using cav-1 small interfering RNA remarkably worsened astrocyte cell damage, while overexpression of cav-1 using a cav-1 CSD peptide attenuated OGD-induced cell apoptosis via Ras/Raf/ERK pathway [161]. This may partly explain why cav-1 inhibits apoptosis in IS.

### Cav-1 and free radicals (Fig. 4)

As discussed in previous sections, ROS and RNS play important roles in IS. Cav-1 has been shown to reduce ROS production in endothelial cells. Mitochondrial ROS production was increased in endothelial cells following cav-1 knockdown. Chen et al. observed that cav-1 inhibited the activity of NADPH oxidases, a major source of cellular ROS [162]. Cav-1-targeted treatments could scavenge reactive species in cancer cells [163]. Src homology 2-containing protein tyrosine phosphatase 2 (SHP-2) provided neuroprotection in a stroke model. Cav-1 protected astrocytes from ROS-induced oxidative stress via SHP-2 activation [164]. According to the aforementioned findings that ROS can induce and/or activate MMPs, we propose that cav-1 protects the BBB from disruption by reduction of ROS and inhibition of MMPs activity.

**Fig. 4:**
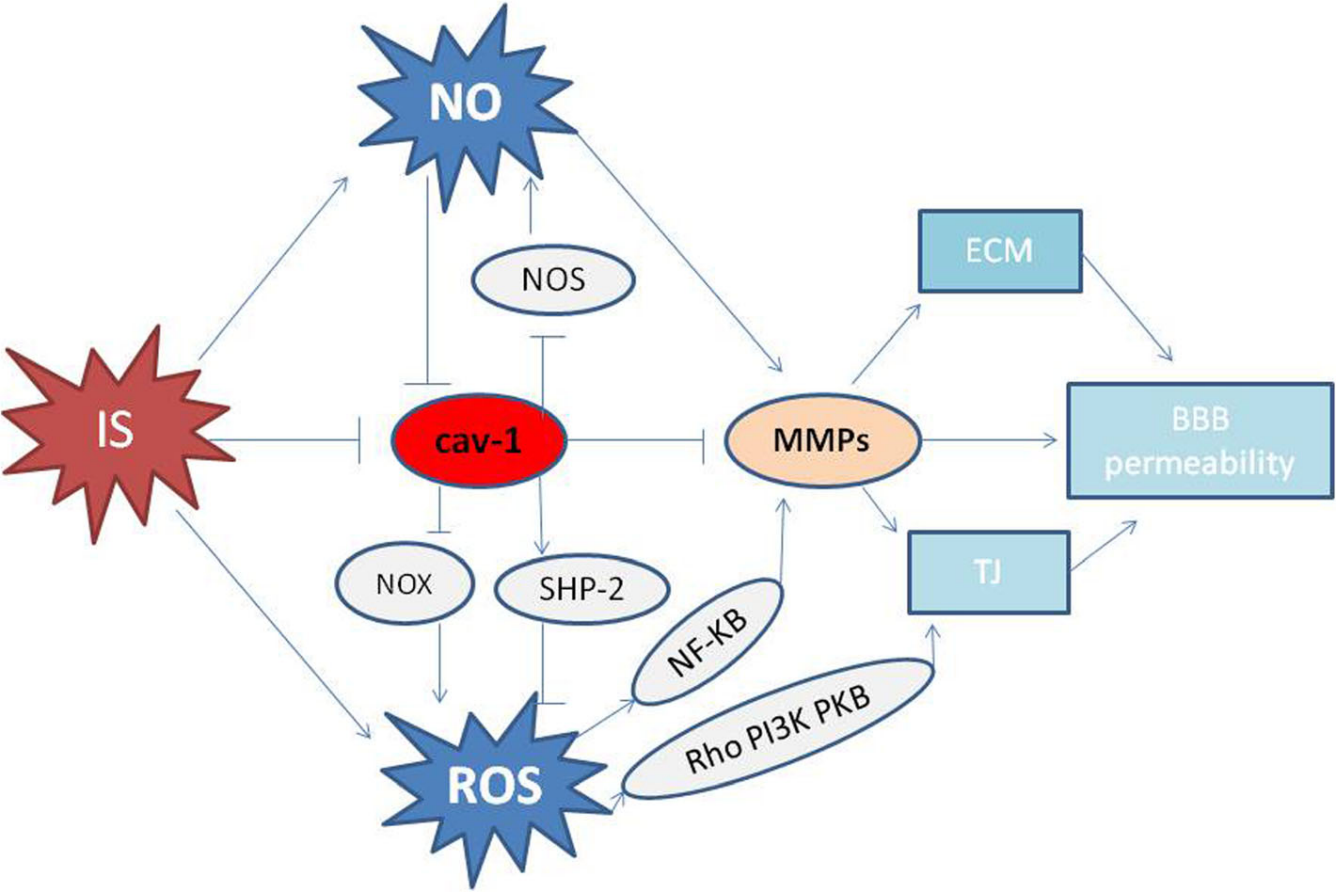
Relationship between cav-1 and free radicals. On one hand, cav-1 directly binds to NOS and decreases NO; on the other hand, NO negatively regulates cav-1 expression. Cav-1 reduces the production of ROS via inhibiting the activity of NADPH oxidases (NOX) and (or) promoting SHP-2 activation. NO and ROS can activate MMP-2 and MMP-9, disrupting TJ and ECM, finally inducing BBB hyperpermeability. Thus, cav-1 can inhibit the activity of MMP2 and MMP9 through reducing NO and ROS. Ischemic stroke (IS), NADPH oxidases (NOX), Src homology 2-containing protein tyrosine phosphatase 2 (SHP-2)

NO can inhibit the expression of cav-1, but cav-1 can inhibit NO production through binding with NO synthase (NOS) and inhibition of cav-1 promotes the activation of NOS and increases NO levels. Glutathione S-transferase (GST) pull-down using purified proteins confirmed that direct binding of cav-1 and NOS occurred between residues 82–101 [165] and that F92 is the key residue to mediate endothelial NO synthase (eNOS) inhibition by cav-1 [166]. High NO concentrations decrease cav-1 abundance and alter its cellular distribution in endothelial cells [167]. NO decreased the expression of cav-1 after IS, and NOS inhibitors prevented the loss of cav-1 in the core and penumbra of the ischemic brain [168]. It is interesting to note that positive regulation of cav-1 by NO is found in tumor cells. The NO donor diethylenetriamine (DETA)/NO upregulated cav-1 in hypoxic human SK-N-MC neuroblastoma cells [33]. Thus, the role of NO in the modulation of cav-1 expression appears to be different in different cell types. Incubation of pure eNOS with peptides derived from the CSD of cav-1 and cav-3 led to inhibition of eNOS, inducible NO synthase (iNOS), and neuronal NO synthase (nNOS) activity [169]. And overexpression of caveolin markedly decreased eNOS enzyme activity in endothelial cells [170]. In addition, delivery of the cav-1 CSD inhibited NO synthesis and inflammation [127]. Loss of cav-1 leads to persistent eNOS activation and high levels of NO in cells, through the loss of cav-1 inhibitory effects on eNOS [171]. It has also been shown that pharmacological blockade or genetic deletion of eNOS prevents many of the physiological changes observed in cav-1 KO mice [172]. Based on the literature, it seems that the interaction of cav-1 and NO partly explains why cav-1 protects BBB integrity from IS. Cav-1 KO mice had increased eNOS activity and NO production; subsequently, NO production directly contributed to MMP activation and BBB disruption. L-NAME preserved the expression of cav-1, inhibited MMPs activity, and reduced BBB permeability.

## Conclusion (Fig. 5)

IS is a major cause of death and long-lasting disability worldwide. In this review, we have introduced several major pathophysiological mechanisms in IS, such as BBB damage, inflammation, and free radicals, and summarized the role of T cells in IS. In addition, we discussed evidence that cav-1 may serve to promote neuroprotection after stroke and also listed some opposing results. Cav-1 OE decreases brain edema in a rat MCAO model, and cav-1 KO increases brain infarction volume. However, some natural active compounds, such as green tea polyphenols, show a protective role in brain ischemia which is correlated with a decrease in cav-1. In angiogenesis, cav-1 negatively regulates endothelial cell proliferation, while promoting differentiation. Cav-1 is necessary for VEGFR-2 to undergo VEGF-dependent activation, but OE of cav-1 inhibits VEGFR-2 activity. As for neurogenesis, treadmill exercise-mediated improved neurological recovery is significantly inhibited when cav-1 is reduced. Cav-1 also enhances pro-survival signaling (NMDAR, TrkB, ERK signaling) and promotes dendritic growth and arborization. It is worth noting that in the hippocampal DG, cav-1 KO mice showed more newborn neurons than the wild-type mice. Cav-1 plays a pro-inflammatory role in endothelial cells, neutrophils, lymphocytes, and microglia, but has an opposing role in macrophages. Cav-1 connects with both the intrinsic and extrinsic apoptotic pathways. Interaction of Fas-ligand and cav-1 provides a molecular basis for the induction of Fas-ligand-dependent cell death. Cav-1 exerts anti-apoptotic action in the intrinsic apoptotic pathway. Using different cell or animal models may help to fully elucidate the role of cav-1 in IS, and we should explore more to answer the controversy.

**Fig. 5:**
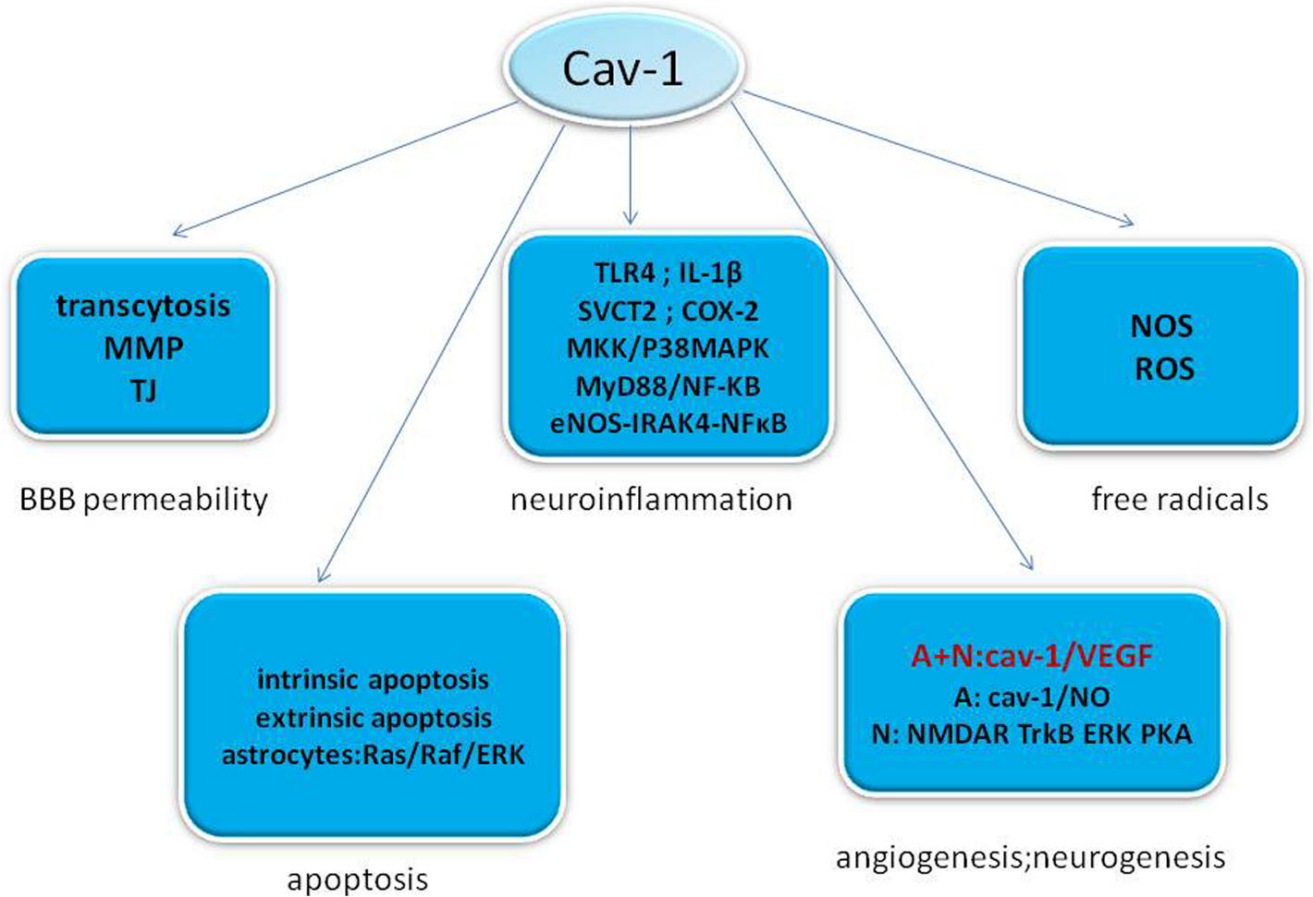
Schematic representation illustrating the role of caveolin-1 in regulating BBB permeability, neuroinflammantion, free radicals, apoptosis, angiogenesis, and neurogenesis and showing some of the signaling pathways coupling caveolae/caveolins with IS. Changing BBB permeability via transcytosis, MMP, and TJ; regulating neuroinflammantion via TLR4, IL-1β, SVCT2, COX2, MKK/P38MAPK, MyD88/NF-ΚB, and eNOS-IRAK4-NFκB pathway; altering free radicals via reducing NOS and ROS; influencing apoptosis through intrinsic apoptotic pathway and extrinsic apoptotic pathway and protecting astrocytes from apoptosis via Ras/Raf/ERK pathway; promoting angiogenesis through cav-1/VEGF and cav-1/NO pathway; and promoting neurogenesis via cav-1/VEGF pathway and neuroplasticity through NMDAR, TrkB, ERK, and PKA pathway. Interleukin-1β (IL-1β), cyclooxygenase-2 (COX-2), mitogen-activated protein kinase (MAPK), reactive nitrogen species (RNS), reactive oxygen species (ROS), extracellular signal-regulated kinase (ERK), vascular endothelial growth factor (VEGF), *N*-methyl-D-aspartate receptor (NMDAR)

## Abbreviations

BBB: Blood–brain barrier
BMECs: Brain microvascular endothelial cells
BTB: Blood-tumor barrier
Cav: Caveolin
CBD: Caveolin-binding motifs
CO: Carbon oxide
COX-2: Cyclooxygenase-2
CSD: Caveolin scaffolding domain
DG: Dentate gyrus
ECM: Extracellular matrix
eNOS: Endothelial NO synthase
ER: Endoplasmic reticulum
ERK: Extracellular signal-regulated kinase
H_2_O_2_: Hydrogen peroxide
HO-1: Heme oxygenase-1
ICAM-1: Intercellular adhesion molecule 1
IL-1: Interleukin-1
iNOS: Inducible NO synthase
IS: Ischemic stroke
KO: Knockout
L-NAME: NG-nitro-L-arginine methyl ester
LPS: Lipopolysaccharide
MCAO: Middle cerebral artery occlusion
MCP-1: Monocyte chemoattractant protein-1
MMPs: Matrix metalloproteases
NMDAR: *N*-methyl-D-aspartate receptor
nNOS: neuronal NO synthase
NSCs: Neural stem cells
OE: Overexpression
OGD: Oxygen-glucose deprivation
PAI-1: Plasminogen activator inhibitor-1
PKA: cAMP-dependent protein kinase
PMN: Polymorphonuclear
RNS: Reactive nitrogen species
ROS: Reactive oxygen species
sHT: Symptomatic hemorrhagic transformation
SVCT2: Plasma membrane sodium-vitamin C cotransporter 2
SVZ: Subventricular zone
TBI: Traumatic brain injury
Tc: Cytotoxic T cells/CD8+ T cells
Th: Helper T cells/CD4+T cells
TJs: Tight junctions
tPA: Tissue plasminogen activator
Tregs: Regulatory cells
TrkB: Tyrosine kinase B receptor
VCAM-1: Vascular cell adhesion molecule-1
VEGF: Vascular endothelial growth factor
VEGFR: Vascular endothelial growth factor receptor
